# Blackflies in the ointment: *O*. *volvulus* vector biting can be significantly reduced by the skin-application of mineral oil during human landing catches

**DOI:** 10.1371/journal.pntd.0007234

**Published:** 2019-04-01

**Authors:** Túllio Romão Ribeiro da Silva, James Lee Crainey, Felipe Arley Costa Pessoa, Yago Vinícius Serra dos Santos, Jordam William Pereira-Silva, Lorena Ferreira de Oliveira Leles, Ana Carolina Vicente, Sérgio Luiz Bessa Luz

**Affiliations:** 1 Instituto Leônidas e Maria Deane/ILMD/FIOCRUZ, Laboratório de Ecologia de Doenças Transmissíveis na Amazônia, 476 Rua Teresina, Adrianópolis, Manaus, Amazonas, Brazil; 2 Programa de Pós-graduação Stricto sensu em Biologia Parasitária do Instituto Oswaldo Cruz (IOC/Fiocruz), Rio de Janeiro, Brazil; 3 Programa de Pós-Graduação em Biologia da Interação Patógeno Hospedeiro (PPGBIO-Interação), Manaus, Amazonas, Brazil; 4 Programa de Pós-Graduação Stricto Sensu em Condições de Vida e Situações de Saúde na Amazônia (PPGVIDA), Manaus, Amazonas, Brazil; 5 Instituto Oswaldo Cruz/IOC/FIOCRUZ, Laboratório de Genética Molecular de Microrganismos, Rio de Janeiro, RJ, Brazil; Yale School of Public Health, UNITED STATES

## Abstract

**Background:**

Standard human landing catches (sHLCs) have historically been a key component of *Onchocerca volvulus* transmission monitoring, but expose health-workers to potentially hazardous vector bites. Novel human-bait-free trapping methods have been developed, but do not always work where they are needed and may not generate *O*. *volvulus* surveillance data that is directly comparable with historic data.

**Methodology:**

Simuliid sHLCs and mineral-oil protected HLCs (mopHLCs) were performed in a rural village of Amazonas state, Brazil. A four-hour direct comparisons of sHLCs and mopHLCs was carried-out using six vector collectors, each of whom used one leg for a sHLC and one for a mopHLC. Two-person collection teams then exclusively performed either mopHLCs or sHLCs for a further set of 12 four-hour collections. Following the completion of all collections, simuliid-bite mark estimates were made from legs used exclusively in sHLCs and legs used exclusively in mopHLCs.

**Principal findings:**

All of the 1669 captured simuliids were identified as the *O*. *volvulus* vector *Simulium oyapockense*. Overall, mopHLC simuliids captured per hour (S/H) rates were lower than those obtained with sHLC trapping (15.5 S/H versus 20 S/H). Direct comparisons of simuliid capture rates found that vector-collectors captured simuliids significantly more efficiently ($\bar{x}$: 20.5 S/H) with mopHLC trapping than with sHLC trapping ($\bar{x}$: 16.4 S/H): P-value = 0.002. MopHLCs performed in isolation were, however, observed to capture vectors less efficiently ($\bar{x}$: 13.4 S/H) than sHLCs performed under similar conditions ($\bar{x}$: 19.98 S/H). All six vector collectors had significantly higher simuliid capture per counted bite mark (SC/CBM) rates using mopHLCs than they were observe to have using sHLCs ($\bar{x}$: 21 SC/CBM versus $\bar{x}$: 1 SC/CBM; p-value = 0.03125).

**Conclusions:**

Vector collectors captured significantly more simuliids per counted bite mark with mopHLCs than with sHLCs. Further investigations into the utility of mopHLCs for onchocerciasis xenomonitoring and beyond are merited.

## Author summary

Standard human landing catches (sHLCs) have historically been used to obtain key *Onchocerca volvulus* transmission data that has helped with the design and monitoring of the WHO´s onchocerciasis control programmes. To avoid the health risks associated with sHLCs, alternative human-bait-free blackfly trapping methods, most of which immobilize and suffocate blackflies with a viscous liquid substance, have been developed. Questions, however, have be raised as to whether these human-bait-free trapping methods generate *O*. *volvulus* transmission data that is directly comparable with historic sHLC data. In this study, we have combined sHLCs with mineral oil vector capture and shown that the skin application of mineral oil can significantly reduce (and possibly eliminate) simuliid biting during HLCs. In direct comparisons, we have shown that mineral oil protected human landing catches (mopHLCs) were more efficient at capturing the *O*. *volvulus* vector *Simulium oyapockense* than sHLCs. We have also shown that mopHLCs, performed in isolation of vector collectors using exposed skin for their trapping, are less efficient than HLCs, but still function well. We believe that mopHLCs represent a promising alternative to sHLCs that merit further testing for their utility in the epidemiological monitoring of onchocerciasis and, indeed, other vector borne diseases as well.

## Introduction

A key component of the WHO´s nascent onchocerciasis elimination programme is the entomological monitoring of *O*. *volvulus* transmission by its blackfly vectors [1,2]. Historically, the African Programme for Onchocerciasis Control (APOC) and Onchocerciasis Elimination Program for the Americas (OEPA) have used human-baited vector collection (HBVC) to calculate infectious bite rates [1–3]. These rates have, in turn, been used to classify regional onchocerciasis endemicity levels and to plan for the cessation of mass drug administration [3–7]. While the epidemiological data generated from such HBVC continues to be valued for such purposes, there is increasing concern about the health risk that the use of this technique poses to vector collectors [8–10].

In order to avoid such health risks, alternative vector capture methods have been developed. Bellec [11] and Bellec-style traps [12,13], which capture ovipositing rather than host-seeking female blackflies, can be used to capture *O*. *volvulus* infected female blackflies [12,13]. However, these traps are not as efficient or convenient as HBVC and questions have been raised as to whether epidemiological data generated from such trapping can be compared directly with historical data obtained from traditional HBVC [9]. Recently, Esperanza Window Traps (EWTs), which use visual and gaseous (CO2) cues to allure host-seeking onchocerciasis vectors, have been developed [8,9]. While epidemiological data collected from these EWTs is more likely to be comparable with that collected with HBVCs, which also target host-seeking female blackflies, these traps have not worked everywhere they have been trialled and therefore probably cannot be used everywhere *O*. *volvulus* entomological monitoring is needed [8,9]. The WHO´s nascent lymphatic and onchocerciasis elimination programme thus urgently needs novel methods of blackfly capture, which do not expose vector collectors to unnecessary risks from vector bites [1,2].

Both the Bellec and EWT trapping methods capture blackflies by immobilizing and suffocating them with a viscous liquid substance that gums-up their delicate wings, legs and respiratory spiracles [8–13]. Here, we have applied mineral oil directly to the legs of vector collectors in order to combine HBVC with mineral oil vector capture. Our work has specifically tested, whether this type of HBVC reduces the number of onchocerciasis vector bites suffered by vector collectors during their collections and therefore whether WHO policy makers should consider recommending this type of vector collection as a substitute for standard human landing catches (sHLCs).

## Materials and methods

### Study site selection

This study was performed in São Gabriel da Cachoeira, which is a rural village of Amazonas state, Brazil. The village is situated deep in the amazon rainforest and close to, but outside, the WHO-recognized Amazonia onchocerciasis focus [14–17]. All of the vector collection sites used for this study are ~600 km South West of the onchocerciasis endemic Yanomami mission post known as Toototobi [17]. São Gabriel da Cachoeira shares many ecological, geophysical and climatic conditions with Toototobi, but, critically, is not thought to have ever had any *O*. *volvulus* transmission [14–17]. Critically too, *Simulium oyapockense*, which is thought to be the principal *O*. *volvulus* vector in Toototobi, is also known to occur abundantly and to bite humans in high numbers in São Gabriel da Cachoeira [15,16]. This village was, thus, considered an ideal location to assess the utility and safety of mopHLC trapping as it was assumed that vector collectors would not be exposed to any unnecessary risk of *O*. *volvulus* infection.

In a pilot study performed in São Gabriel da Cachoeira between the 25th and the 30th of September (inclusive), in which 4,781 simuliids were collected from five sites, all but one of the simuliids collected were identified as *S*. *oyapockense*. Although the one non-*S*. *oyapockense* collected in the pilot study (which was identified as *Simulium ochraceum*) is also a known vector of *O*. *volvulus*, we decided to exclude the collection site where it was captured in an attempt to collect data that was directly attributable to the behaviour of just one species of blackfly vector (*S*. *oyapockense*). We chose to exclude another site used in the pilot study because it was the least productive of the remaining four. Blackfly vector collections for this study were, thus, performed at three sites within São Gabriel da Cachoeira, each more than 0.5 km apart: Collection site (1): named Comunidade Areal: 0°8'60''S/66°57'7.2''W; collection site (2): named “Porto de Camanaus”: 0°8'56''S/66°56'8.79''W, and collection site (3): named “Casa de Camanaus”: 0°8'51''S/66°56'23.23''W.

### Vector collector recruitment

In order to recruit vector collectors without pre-existing simuliid bites on their legs, six Manaus residents were selected to participate in this study. While Manaus, like São Gabriel da Cachoeira, is also situated in Amazons state (Brazil) and is also surrounded by the Amazon rainforest, it is a city of more than two million inhabitants and suffers from high levels of watercourse pollution, which prevents simuliid larval breeding and thus simuliid adult biting in the area occurs only rarely (if at all) [18–20].

### Vector capture: The mopHLC procedure

MopHLCs were performed by vector collectors applying approximately 50 ml of pharmaceutical-grade mineral oil to the skin surface of one of their legs. Vector collectors were asked to apply the mineral oil (which was sourced from a local chemist) evenly and as a thick continuous layer. They were also asked to avoid trying to rub the oil into their skin (as one might with a sun cream) so that the mineral oil formed a continuous barrier between the vector collectors´ skin and the open-air. The Rioquímica (São Paulo, Brazil) mineral oil used for this study is a typical pharmaceutical-grade mineral oil, which can be cheaply and easily purchased from chemists throughout Brazil. It is a colourless, odourless mixture of liquid hydrocarbons, produced from petroleum distillation. During mopHLC collections, blackflies landing on mineral oil protected legs were quickly immobilised by it and then transferred manually with watchmakers´ forceps from the oil to a glass collection tube filled with 100% ethanol.

### Vector capture: The sHLC procedure

Blackflies landing on the unprotected legs were collected directly in ethanol using a sHLC collection procedure that is widely practiced in the area and elsewhere in the world [10,21]. For this procedure, the vector collector touched the ~1.5 cm diameter mouth of a 6 ml (bijou-style) glass collection tube (brimming with 80–100% ethanol) to a patch of their skin that a female blackfly that had just landed on [21]. Typically, during this study blackflies did not move before they came into contact with the tube´s ethanol and would subsequently sink to the bottom of the tube shortly after they had. Occasionally, blackflies would attempt to take-off before being collected; however, many of these blackflies were also collected as they often flew directly into the collection tube.

### Vector capture: Experimental time-line and overview

The efficiency and safety of the mopHLCs and sHLCs vector capturing techniques was compared using a set of 24 four-hour vector collections performed daily between 8 am and 12 noon from the 26th to the 28th of October 2017. The 24 collections used one vector collector leg each and were performed in two-person teams at the three collections sites, which are described in detail in the ‘study site selection” section above. The three vector collection teams used for this study were composed of the following vector collectors: JWPS and CAC, team 1; JLC and TTRS, team 2, and FACP and YVSS, team 3. Collection team 1 collected at collection site 1 on the 26th of October 2017; at collection site 2 on the 27th and at collection site 3 on the 28th. Collection team 2 collected at site 2 on the 26th; at site 3 on the 27th and at site 1 on the 28th. Collection team 3 collected at site 3 on the 26th; at site 1 on the 27th and at site 2 on the 28th.

Prior to the initiation of the study, all six vector collectors were asked to randomly select one leg to use exclusively for all of their mopHLC trapping and told to use their other leg for all of their sHLC trapping. Vector collectors were also instructed not to tell any of the other vector collectors which leg they had chosen to use for either type of trapping. This was done so as to reduce the risk of observer bias during the bite-counting stage of this study (see below). To protect the vector collectors from the risk of sunburn, vector collectors were also asked to apply factor 50 sunscreen to their legs approximately one hour before beginning their collections for every day of the study.

### Vector capture on the 26th of October: Simultaneous mopHLCs and sHLCs

On the 26th of October 2017 experiments designed to directly compare between mopHLCs and sHLCs were carried out. For this, a set of 12 four-hour vector collections were performed by our three two-person vector collector teams. During these collections, each of our six vector collector used one of their legs for mopHLC vector capture and one of their legs for sHLC vector capture.

### Vector capture on the 27th and 28th of October 2017: mopHLC-only and sHLC-only vector collections

In order to assess the efficiency of mopHLCs performed in isolation of exposed human legs, on the 27th and 28th of October our vector collector teams were asked to perform exclusively one type of vector collection: either mopHLCs or sHLCs. During the course of these two days a total of 12 vector collections (in which a single vector collector used one leg to collect vectors) were performed by our three vector collector teams: eight mopHLCs and four sHLCs. Four of these mopHLCs collections were performed on the 27th of October by vector collector teams 1 and 3 and four were performed on the 28th by vector collect teams 2 and 3. Vector collector teams 1 and 2, performed sHLCs (in isolation of mopHLCs) on the 28th and 27th of October 2017, respectively.

### Vector identification

Blackflies collected during the course of this study, by both mopHLCs and sHLCs, were all identified as *S*. *oyapockense* using morphological keys and information provided in Shelley et al. 2010 [15] and Hamada et al. 2015 [16].

### Bite counting: Rationale and overview

While the vector collection data above was sufficient to compare the capture efficiencies of mopHLCs and sHLCs, it was necessary to estimate the number of vector bites that vector collectors suffered during their collections in order to compare their safety. For this reason, estimates of the number of bites each of our vector collectors suffered during their collections were made and used (together with our collection data) to calculate estimates of the number of simuliids that each vector collector captured for each bite they suffered. In recognition that the form of simuliid bite marks varies over time and thus that the reliability with which they can be discriminate from other types of skin blemish also varies, we made two estimates of the number of bite marks each vector collector suffered. One set of estimates was made using bite counts taken immediately after all of the vector collections were completed (on the 28th of October 2017) and one was based on bite counts that were made two days later (on the 30th of October).

### Bite counting: The procedure

**Fig 1:**
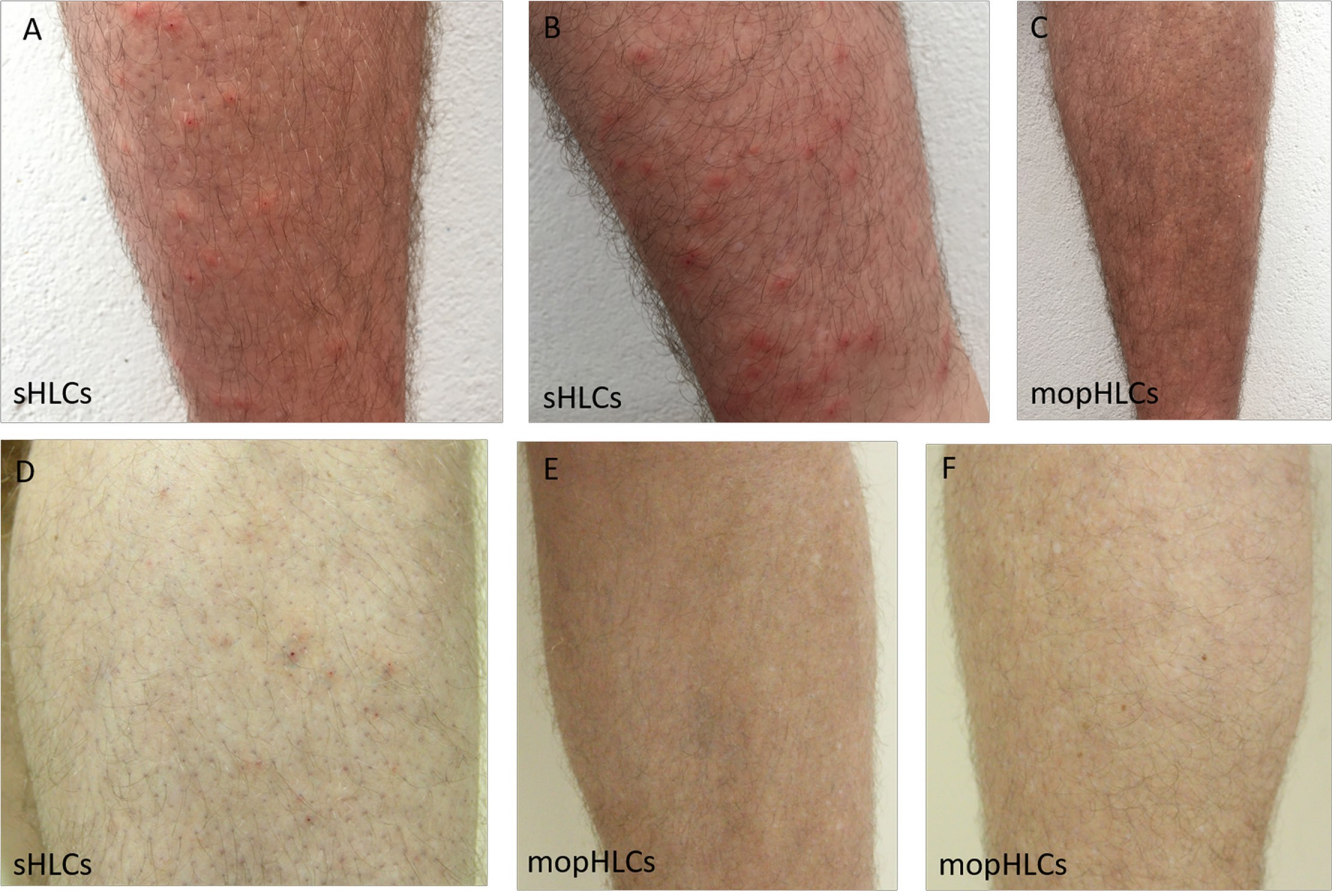
Shows close-up photographs of the legs of vector collector JLC, taken on the 28th of October 2017 (panels A to C) and the 30th of October 2017 (panels D to F). Panels A, B and D are photographs of JLC´s right leg, which was exclusively used for sHLC trapping; panels C, E and F are of JLC´s left leg, which was used exclusively for mopHLC trapping. In panels A, B and D simuliid bite marks can be identified by their distinctive deep-red pin-head-sized scab mark, which are formed from the wound simuliids create to pool-feed [23]. Many of the bite marks shown in panels A and B can be seen to be ringed by a pinkish (and slightly raised) disc of skin about 5 mm in diameter. While some of the bite marks visible in panel D are also ringed by pinkish skin, the bite marks are less pronounced.

**Fig 2:**
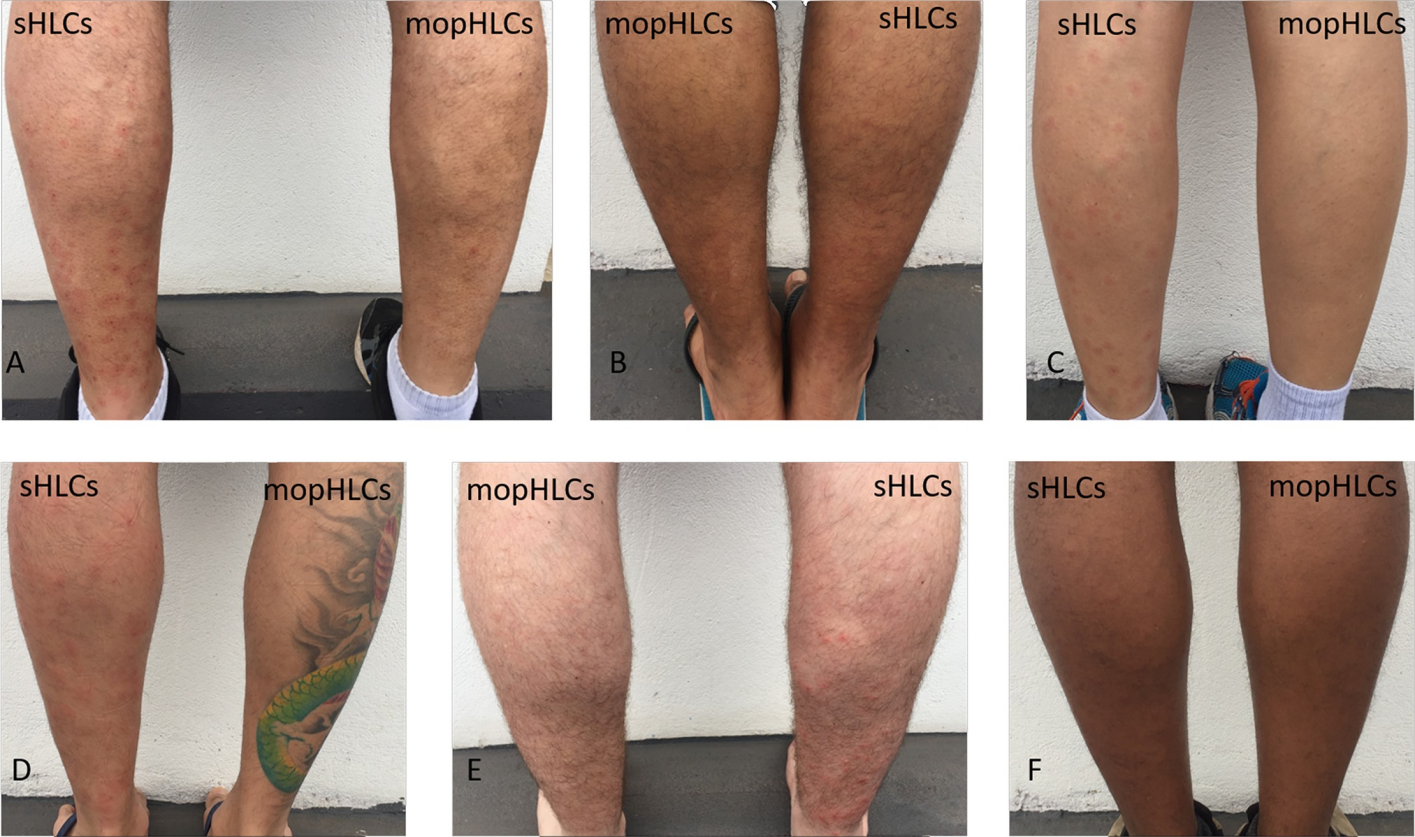
Shows photographs of the legs of our six vector collectors after our final day of simuliid collection (the 28th of October 2017), with the trapping method used indicated. Simuliid bite mark count data from the day these photographs were taken can be seen in Table 1. The legs of our vector collectors are show in the following sequence: FACP (panel A); JMPS (panel B); CAC (panel C); TRRS (panel D); JLC (panel E) and YVSS (panel F).

**Fig 3:**
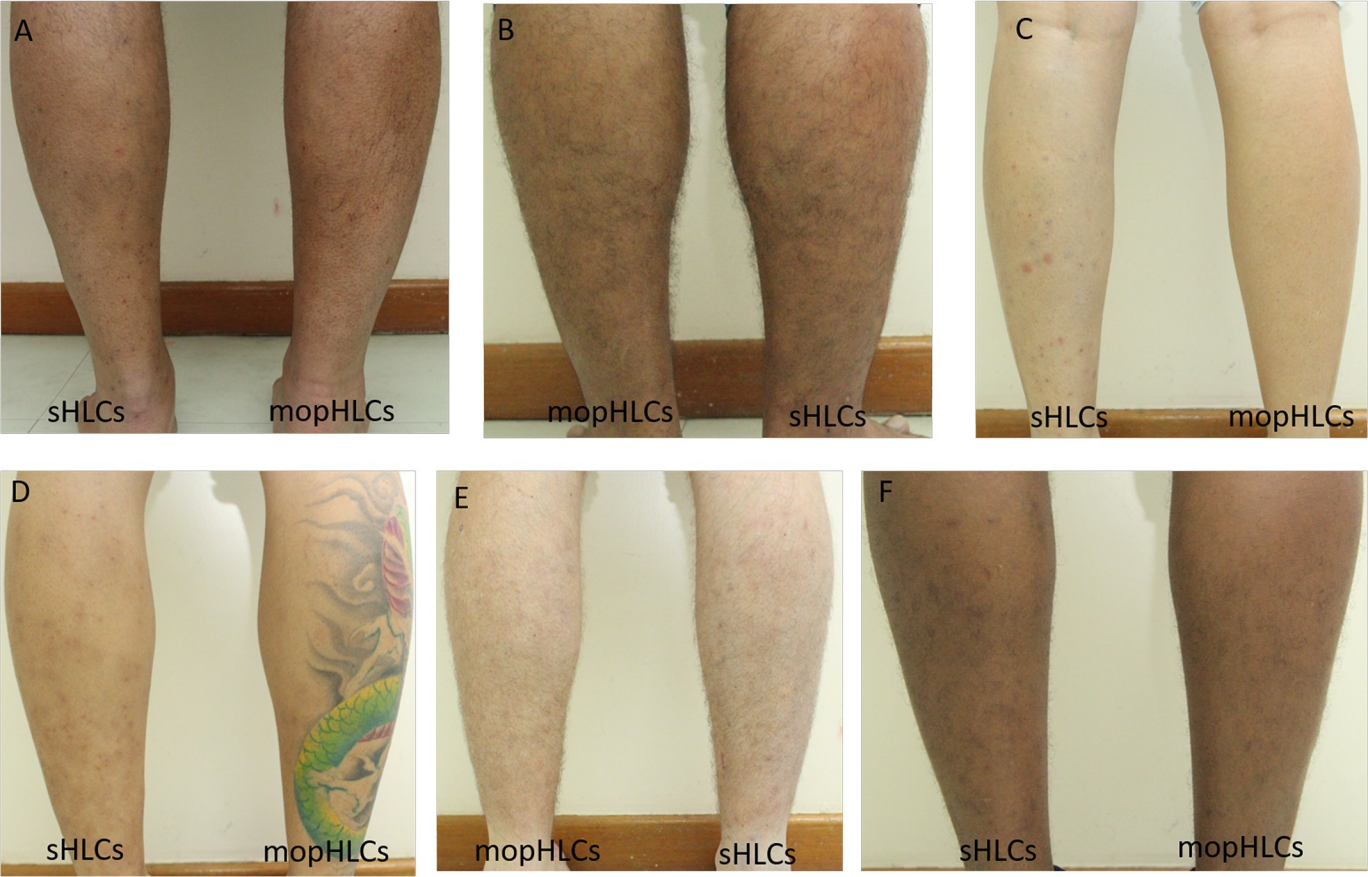
Shows photographs taken at the end of our “blind” bite-mark counting session on the 30th of October 2017. Vector collector legs (panels A-F) appear in the same sequence as they do in Fig 2 and are also labelled in the same way. The bite count data obtained on the day these photos were taken are shown in Table 2.

Prior to the initiation of bite counting and on both bite-counting days, bite counters were trained how to identify a simuliid bite-mark using photographs (similar to those shown in Figs 1–3). Bite counters were then asked to circle all of the simuliid bite marks they could see on each of our vector collector´s legs and then count-off the bite marks. After each bite counter had completed their counting, they wiped-clean the pen-marks on the vector collectors legs with 100% ethanol. A subsequent bite-count of a vector collectors legs was only completed once all traces of the marker pen had been cleaned away. To minimise the impact of our vector collectors suffering blackfly bites out-side the study period, our vector collectors were asked to use long trousers while not collecting blackflies and to declare any simuliid bite marks they had acquired prior to the initiation of the study (so they could be discounted from the study). Two of our vector collectors declared the existence of simuliid bites on their legs (FACP and JWPS) prior to the initiation of the study and had these bite-marks excluded from their analysis; the other four had no visual sign of simuliid bite-marks on the legs prior to the initiation of the study.

### Bite-mark counting on the 28th of October 2017: Fresh bite marks and semi-blind bite counters

The first set of bite counts were taken in São Gabriel da Cachoeira immediately after the completion of the third day of vector collection on the 28th of October. These bite counts were made when simuliid bite-marks were still fresh and at their most visible (see Fig 1) and were performed by our six study vector collectors. To minimise the impact of observer bias, vector collectors did not count bite marks on their own legs or on the legs of their vector collecting partner. This meant that on the 28th of October four bite counts were taken for each leg of each of our vector collectors. Although the bite-counters used in this part of our study did not know whether the legs they were counting bites from had been used for mopHLCs or sHLCs they did know the objectives of our study and for this reason we have classified these bite counts as “semi-blind”. The raw data from these bite counts is shown in Table 1.

**Table 1:** Simuliid trapping efficiency and safety data calculated from fresh bite marks and semi-blind bite counters.

	Date	Simuliid capture		Bite estimates												SC/CBM	SC/CBM	
		mopHLC	sHLCs	mopHLC				sHLC								mopHLC	sHLC	p-value
				1	2	3	4	AV	SD	1	2	3	4	AV	SD			
**VC: FACP**	**26/10/2018**	**115**	**45**	-	-	-	-	-	-	-	-	-	-	**-**	**-**	**-**	**-**	**-**
	**27/10/2018**	**72**	**-**	-	-	-	-	**-**	**-**	-	-	-	-	**-**	**-**	**-**	**-**	**-**
	**28/10/2018**	**52**	**-**	-	-	-	-	**-**	**-**	-	-	-	-	**-**	**-**	**-**	**-**	**-**
	**28/10/2018**			4	1	1	4	**2.5**	**1.7**	162	197	222	111	**173**	**48.1**	**95.6**	**0.26**	**0.03125**
**VC: JLC**	**26/10/2018**	**72**	**49**	-	-	-	-	**-**	**-**	-	-	-	-	**-**	**-**	**-**	**-**	**-**
	**27/10/2018**	**-**	**54**	-	-	-	-	**-**	**-**	-	-	-	-	**-**	**-**	**-**	**-**	**-**
	**28/10/2018**	**42**	**-**	-	-	-	-	**-**	**-**	-	-	-	-	**-**	**-**	**-**	**-**	**-**
	**28/10/2018**			0	0	0	0	**0**	**0**	170	198	173	112	**163.25**	**36.3**	**-**	**0.63**	**0.03125**
**VC: TRRS**	**26/10/2018**	**164**	**71**	-	-	-	-	**-**	**-**	-	-	-	-	**-**	**-**	**-**	**-**	**-**
	**27/10/2018**	**-**	**83**	-	-	-	-	**-**	**-**	-	-	-	-	**-**	**-**	**-**	**-**	**-**
	**28/10/2018**	**123**	**-**	-	-	-	-	**-**	**-**	-	-	-	-	**-**	**-**	**-**	**-**	**-**
	**28/10/2018**			8	13	0	4	**6.25**	**5.6**	229	172	292	227	**230**	**49.1**	**45.92**	**0.66**	**0.03125**
**VC: JWPS**	**26/10/2018**	**65**	**64**	-	-	-	-	**-**	**-**	-	-	-	-	**-**	**-**	**-**	**-**	**-**
	**27/10/2018**	**8**	**-**	-	-	-	-	**-**	**-**	-	-	-	-	**-**	**-**	**-**	**-**	**-**
	**28/10/2018**	**-**	**202**	-	-	-	-	**-**	**-**	-	-	-	-	**-**	**-**	**-**	**-**	**-**
	**28/10/2018**			0	0	0	0	**0**	**0**	89	143	142	107	**120.25**	**26.7**	**-**	**2.21**	**0.03125**
**VC: YVSS**	**26/10/2018**	**21**	**67**	-	-	-	-	**-**	**-**	-	-	-	-	**-**	**-**	**-**	**-**	**-**
	**27/10/2018**	**50**	**-**	-	-	-	-	**-**	**-**	-	-	-	-	**-**	**-**	**-**	**-**	**-**
	**28/10/2018**	**28**	**-**	-	-	-	-	**-**	**-**	-	-	-	-	**-**	**-**	**-**	**-**	**-**
	**28/10/2018**			3	3	1	0	**1.75**	**1.5**	107	102	101	103	**103.25**	**2.6**	**56.57**	**0.64**	**0.03125**
**VC: CAC**	**26/10/2018**	**57**	**103**	-	-	-	-	**-**	**-**	-	-	-	-	**-**	**-**	**-**	**-**	**-**
	**27/10/2018**	**0**	**-**	-	-	-	-	**-**	**-**	-	-	-	-	**-**	**-**	**-**	**-**	**-**
	**28/10/2018**	**-**	**62**	-	-	-	-	**-**	**-**	-	-	-	-	**-**	**-**	**-**	**-**	**-**
	**28/10/2018**			4	1	0	4	**2.25**	**2.3**	124	104	72	60	**90**	**29.3**	**25.33**	**1.83**	**0.03125**
	**Study averages**							**2.125**	**1.85**					**146.625**	**32.01**	**37.23**	**1.03**	

Shows all the simuliid vector capture data from the three days of our study and bite count data collected by semi-blind bite counters taken on the 28th of October 2017. Abbreviations not used or defined elsewhere are as follows: “VC” for vector collector; “SD” for standard deviation and “AV” for average. The symbol “$\bar{x}$” is used to indicate the sample mean obtained from our bite-count estimates. The p-values quoted in the final column of the table indicate the significance of the difference observed between mopHLC SC/CBMs and sHLC SC/CBMs.

### Bite-mark counting on the 30th of October 2017: Mature bite marks and fully blind bite counters

The second set of bite counts was taken in Manaus using only “fully blind” simuliid bite counters. These bite counters were not only unaware of which legs the vector collectors had used for mopHLC and sHLCs, but were, in fact, entirely ignorant of all aspects of our study´s design and objectives. A set of ten such bite counters were used on the 30th October to obtain a total of six bite mark estimates for each of the legs of each of our six vector collectors. The raw data from these bite counts is shown in Table 2.

**Table 2:** Simuliid trapping efficiency and safety data calculated from mature bite marks and fully-blind bite counters.

	Date	Simuliid Capture	Bite estimates																SC/CBM	SC/CBM	
		mopHLC	sHLCs	mopHLC						sHLC										mopHLC	sHLC	p-value
** **	** **	** **	** **	**1**	**2**	**3**	**4**	**5**	**6**	**AV**	**SD**	**1**	**2**	**3**	**4**	**5**	**6**	**AV**	**SD**	** **	** **	** **
**VC: FACP**	**26/10/2018**	**115**	**45**	-	-	-	-	-	-	**-**	**-**	-	-	-	-	-	-	-	-	-	-	-
	**27/10/2018**	**72**	**-**	-	-	-	-	-	-	**-**	**-**	-	-	-	-	-	-	-	-	-	-	-
	**28/10/2018**	**52**	**-**	-	-	-	-	-	-	**-**	**-**	-	-	-	-	-	-	-	-	-	-	-
	**30/10/2018**			30	22	0	2	13	14	**13.5**	**11.5**	123	105	122	92	172	154	**128**	**30**	**17.7**	**0.4**	**0.03125**
**VC: JLC**	**26/10/2018**	**72**	**49**	-	-	-	-	-	-	**-**	**-**	-	-	-	-	-	-	**-**	**-**	**-**	**-**	**-**
	**27/10/2018**	**-**	**54**	-	-	-	-	-	-	**-**	**-**	-	-	-	-	-	-	**-**	**-**	**-**	**-**	**-**
	**28/10/2018**	**42**	**-**	-	-	-	-	-	-	**-**	**-**	-	-	-	-	-	-	**-**	**-**	**-**	**-**	**-**
	**30/10/2018**			14	2	0	5	4	3	**4.6**	**4.9**	123	116	171	126	90	132	**126.3**	**26.3**	**27.8**	**0.8**	**0.03125**
**VC: TRRS**	**26/10/2018**	**164**	**71**	-	-	-	-	-	-	**-**	**-**	-	-	-	-	-	-	**-**	**-**	**-**	**-**	**-**
	**27/10/2018**	**-**	**83**	-	-	-	-	-	-	**-**	**-**	-	-	-	-	-	-	**-**	**-**	**-**	**-**	**-**
	**28/10/2018**	**123**	**-**	-	-	-	-	-	-	**-**	**-**	-	-	-	-	-	-	**-**	**-**	**-**	**-**	**-**
	**30/10/2018**			10	11	0	5	13	14	**8.8**	**5.3**	158	200	225	241	211	186	**203.5**	**29.4**	**32.6**	**0.8**	**0.03125**
**VC: JWPS**	**26/10/2018**	**65**	**64**	-	-	-	-	-	-	**-**	**-**	-	-	-	-	-	-	**-**	**-**	**-**	**-**	**-**
	**27/10/2018**	**8**	**-**	-	-	-	-	-	-	**-**	**-**	-	-	-	-	-	-	**-**	**-**	**-**	**-**	**-**
	**28/10/2018**	**-**	**202**	-	-	-	-	-	-	**-**	**-**	-	-	-	-	-	-	**-**	**-**	**-**	**-**	**-**
	**30/10/2018**			14	4	0	7	14	25	**10.7**	**8.9**	155	77	214	203	178	191	**169.6**	**49.8**	**6.8**	**1.6**	**0.03125**
**VC: YVSS**	**26/10/2018**	**21**	**67**	-	-	-	-	-	-	**-**	**-**	-	-	-	-	-	-	**-**	**-**	**-**	**-**	**-**
	**27/10/2018**	**50**	**-**	-	-	-	-	-	-	**-**	**-**	-	-	-	-	-	-	**-**	**-**	**-**	**-**	**-**
	**28/10/2018**	**28**	**-**	-	-	-	-	-	-	**-**	**-**	-	-	-	-	-	-	**-**	**-**	**-**	**-**	**-**
	**30/10/2018**			1	10	0	21	5	9	**7.7**	**7.7**	80	103	117	108	92	114	**102.3**	**14.1**	**12.8**	**0.7**	**0.03125**
**VC: CAC**	**26/10/2018**	**57**	**103**	-	-	-	-	-	-	**-**	**-**	-	-	-	-	-	-	**-**	**-**	**-**	**-**	**-**
	**27/10/2018**	**0**	**-**	-	-	-	-	-	-	**-**	**-**	-	-	-	-	-	-	**-**	**-**	**-**	**-**	**-**
	**28/10/2018**	**-**	**62**	-	-	-	-	-	-	**-**	**-**	-	-	-	-	-	-	**-**	**-**	**-**	**-**	**-**
	**30/10/2018**			0	0	0	0	7	5	**2**	**3.2**	91	73	84	82	128	102	**93.3**	**19.6**	**28.5**	**1.8**	**0.03125**
	**Study averages**									**7.9**	**7.8**							**137.2**	**28.2**	**21**	**1**	

Shows all the simuliid vector capture data from the three days of our study and bite estimate data collected by our “blind” bite-counters taken on the 30th of October 2017. The abbreviations used in this table are the same as those used for Table 1.

### Statistical analysis

Differences between the number of simuliids captured per counted bite mark (SC/CBM) obtained using sHLC and mopHLC trapping were tested for statistical significance using Wilcoxon rank sum tests. Observed differences in the efficiency of collections (i.e the number of simuliids captured per hour) in direct comparison between sHLCs and mopHLCs were tested for significance using a paired Wilcoxon signed rank test. Observed differences in the efficiency of simuliid collection with mopHLCs performed in isolation of sHLCs and various kinds of sHLCs were tested for significance using Wilcoxon sum rank tests [22]. All statistical analysis was implemented in the statistical analysis program R (version 3.4.2) [23].

### Ethical statement

Vector capture was performed following a protocol approved by the research ethics committee of the Fundação Osvaldo Cruz-FIOCRUZ/IOC (approval number CAAE: 41678515.1.0000.5248) and followed a similar approach to that described by Shelley et al. [20]. All six vector collector participants recruited to the study were adults (over the age of 23) and had the experiment explained and its objective explained to them. All six participates provided written consent for their participation in the study. All six vector collectors also provided their consent to be identified in the figures used in this manuscript.

## Results

### Simuliid vector capture and identification

Both mopHLCs and sHLCs proved highly successful methods of simuliid capture at all three collection sites in São Gabriel da Cachoeira. In total 1,669 blackflies were captured for this study: 869 with mopHLC and 800 with sHLC, all of which were identified as *S*. *oyapockense* (Tables 1 and 2).

### Simuliid vector capture efficiency using mopHLCs and HLCs simultaneously

**Fig 4:**
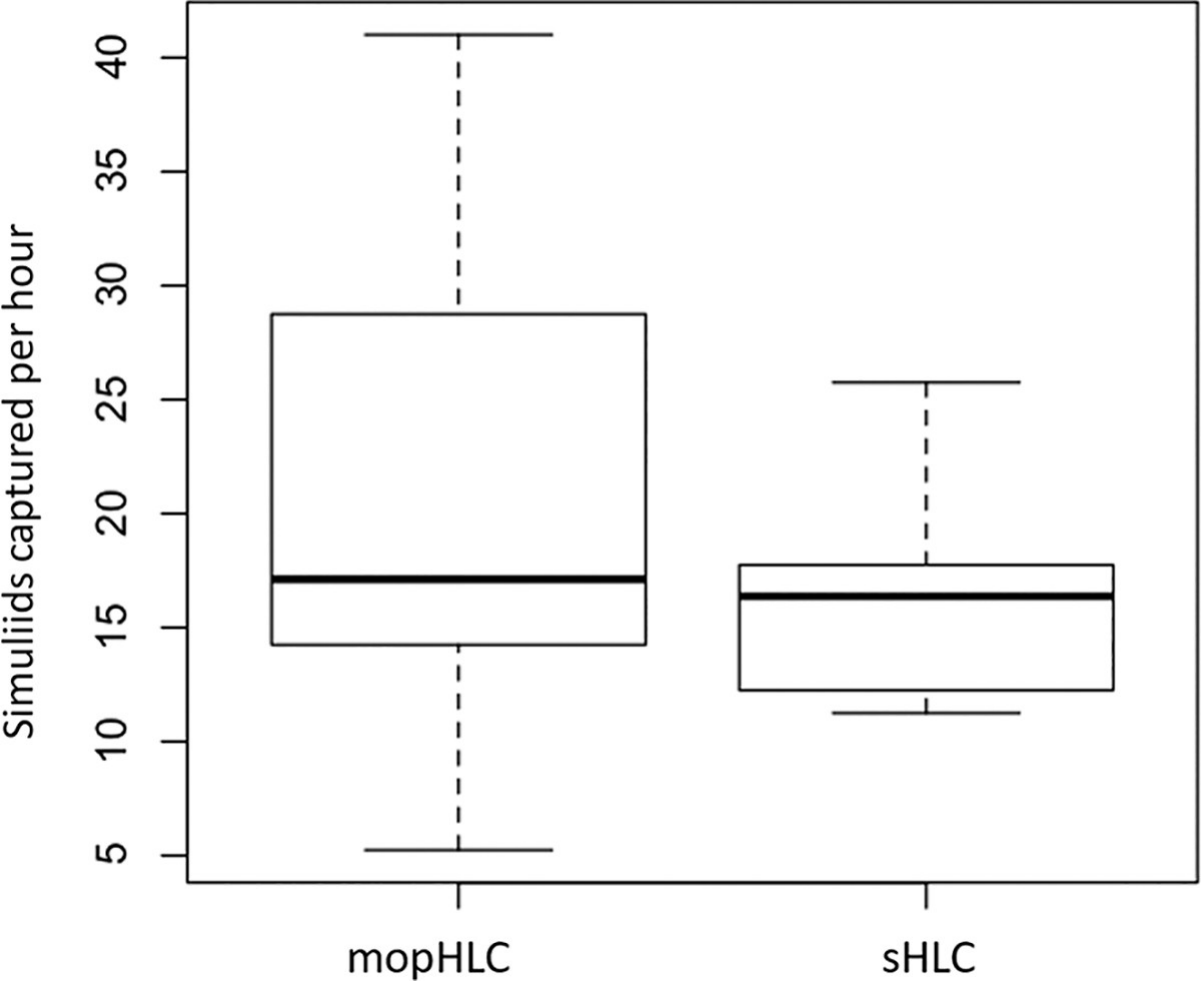
Shows a trapping efficiency comparison between mopHLCs and sHLCs. The raw data used to prepare these box plots is shown in Tables 1 and 2 and derives from six vector collectors who performed both collection techniques simultaneously on the 26th of October 2017. The Y-axis of the graph shows the efficiency with which simuliids were trapped in units of “simuliids captured per hour”.

Simuliid vectors were captured more efficiently with mopHLCs than they were with sHLCs when the two techniques were performed simultaneously on the 26th of October 2017 (Fig 4 and Tables 1 and 2). Vector collectors were calculated to capture between 5.25 and 28.75 simuliids per hour (S/H) using mopHLCs and between 11.25 and 25.75 S/H using sHLCs. On average simuliids were captured at a rate of 20.5 S/H ($\bar{x}$) using mopHLC and at a rate of 16.4 S/H ($\bar{x}$) by sHLC trapping. The difference between the two collection techniques´ capture rates was found to be significant using a paired Wilcoxon signed rank test: P-value = 0.002.

### Simuliid vector capture efficiency using mopHLCs in isolation of sHLCs

**Fig 5:**
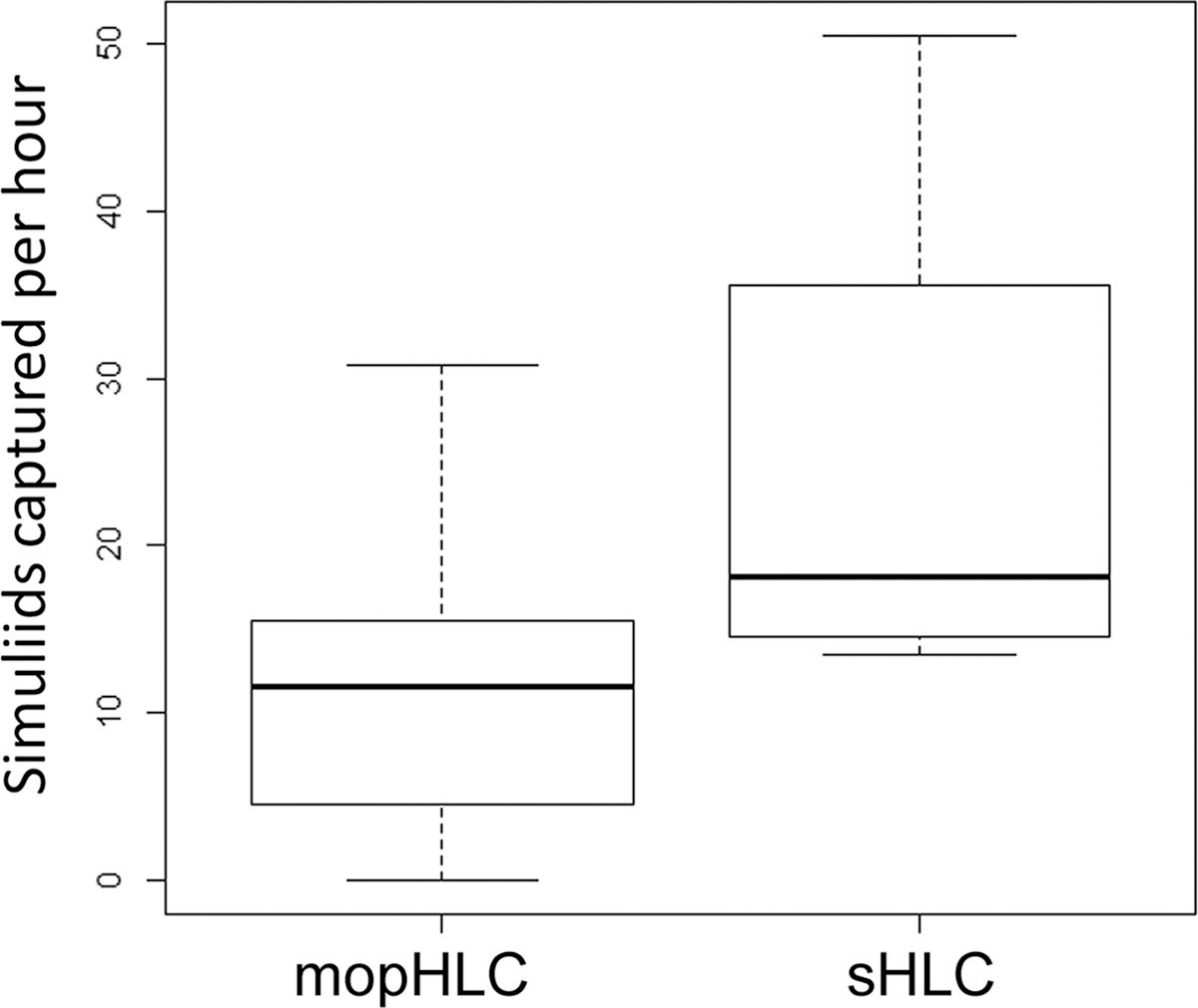
Shows a capture efficiency comparison between sHLCs and mopHLCs performed in isolation of sHLCs. These box plots were prepared from simuliid capture data collected from the eight mopHLCs and four sHLCs performed between the 27th and 28th of October 2017. The Y-axis of the graph indicates the efficiency with which each technique traps simuliids in units of “simuliids captured per hour”.

**Fig 6:**
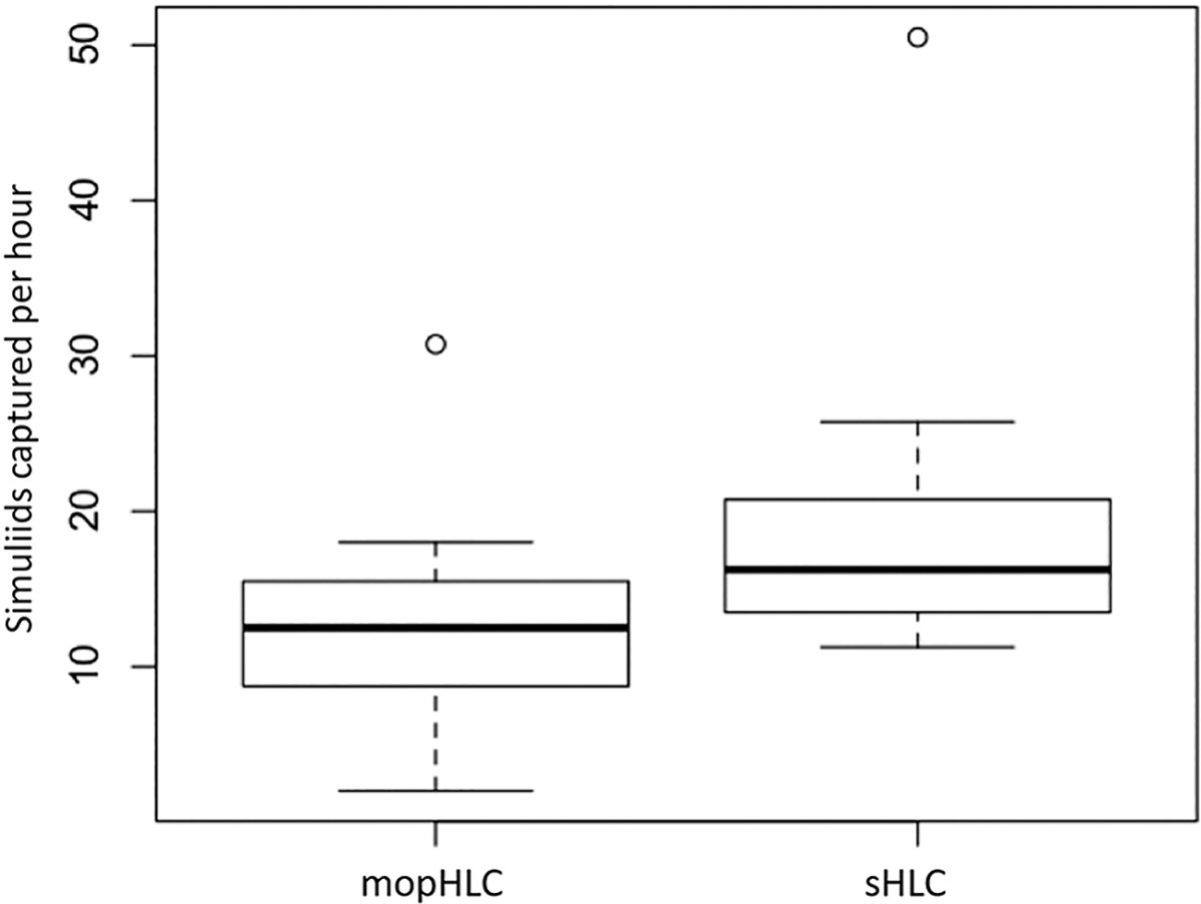
Shows a capture efficiency comparison between sHLCs and mopHLCs performed in isolation of sHLCs. The box plots uses data from the eight mopHLCs (performed between the 27th and 28th of October 2017) and 10 sHLCs performed between the 26th and 28th of October 2017. The Y-axis of the graph indicates the efficiency with which each technique traps simuliids in units of: “simuliids captured per hour”.

Simuliid vectors were captured less efficiently with mopHLCs than they were with sHLCs when mopHLCs were performed in the absence of vector collectors performing sHLCs and thus when they were performed in the absence of exposed leg skin (Fig 5 and Table 1). Fig 5 shows a comparison of the eight mopHLCs and the four sHLCs capture rates calculated from collections performed on the 27th and 28th of October. In contrast to what was observed in direct comparisons (performed on the 26th of October), the eight mopHLCs performed in isolation of sHLCs were observed to capture simuliids significantly less efficiently ($\bar{x}$: 11.72 S/H) than the four sHLCs ($\bar{x}$: 25.06 S/H) performed over the same period (P = 0.002165, Wilcoxon sum rank test). A significant difference (P = 0.002165, Wilcoxon sum rank test) was also found when these eight mopHLCs (performed in isolation of exposed leg skin) were compared with the collection data obtained from all 12 sHLCs used in this study i.e when sHLC data from the 26th was included in the analysis (Fig 6).

### Estimating the number of simuliid bites suffered by vector collectors using mopHLC and sHLC vector capture techniques

Fig 2 shows the state of the legs of our vector collectors immediately after the completion of collections and thus at the time when our “semi-blind” bite counters conducted their fresh bite counts. Fig 3 shows the state of our vector collectors´ legs on the day that our “fully blind” bite-mark counters made their bite mark counts. Using mopHLC vector capture, vector collectors were estimated to have suffered between zero and 6.25 ($\bar{x}$) simuliid bites by our semi-blind counters (see Table 1) and between two ($\bar{x}$) to 13.5 ($\bar{x}$) bites by our fully blind counters (see Table 2). Whereas using sHLC vector capture, our vector collectors were estimated to have suffered between 90 ($\bar{x}$) and 173 ($\bar{x}$) bite marks by our semi-blind bite counters (Table 1) and between 93 ($\bar{x}$) and 203 ($\bar{x}$) by our bite marks by our fully blinded bite counters (Table 2).

### Estimating the number of simuliids captured per bite suffered using mopHLCs and sHLCs

**Fig 7:**
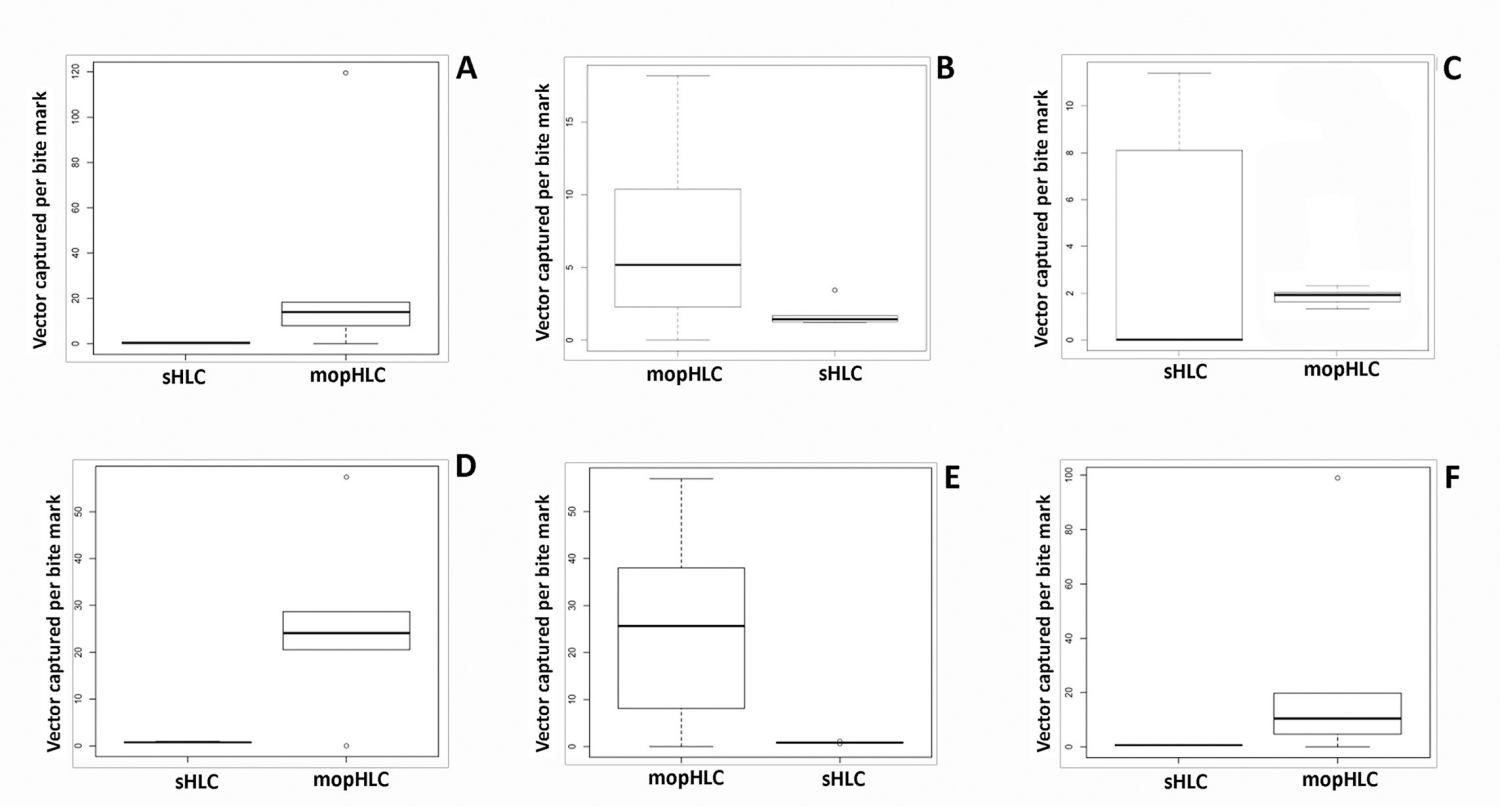
Shows box plots comparing SC/CBMs calculated for mopHLC and sHLC trapping. The S/CBM box plots were prepared individually from each of our six vector collectors collection data. Graphs A-F correspond to data taken from the vector collectors´ legs shown in Figs 2 and 3 (plates A-F) and are labelled in the same way. The raw collection data used to prepare these graphs is shown in Table 2.

For each of our six vector collectors, the number of simuliids captured per counted bite mark counted (SC/CBM) was calculated separately for both their mopHLC and sHLC collections (Tables 1 and 2). The set of six SC/CBMs calculated from the fully blind bite count data are shown graphically in Fig 7. Using this data, mopHLCs were calculated to have SC/CBMs of between 6.8 and 32.6 [$\bar{x}$: 21]; whereas sHLCs were calculated to have SC/CBMs of between 0.4 and 1.8 [$\bar{x}$: 1] (Table 2). Using the semi-blind count data, mopHLCs were calculated to have SC/CBMs of between 25.33 and 95.6 [$\bar{x}$: 37.23]; whereas sHLCs were calculated to have SC/CBMs of between 0.26 and 2.21 [$\bar{x}$: 1.03] (Table 1). As can be seen in Tables 1 and 2, Wilcoxon rank sum tests showed that all six vector collectors had SC/CBMs that were all significantly higher for their mopHLC collections than for their sHLCs collections (p-values: ≤ 0.03125), regardless of which set of bite estimates were used to calculate the SC/CBMs. Our results therefore show that our vector collectors that applied mineral oil to their skin during human landing catches captured significantly more *S*. *oyapockense* for each bite they suffer than they did using sHLCs. In our experiments, thus, mopHLCs were seen to be significantly safer than sHLCs for onchocerciasis vector trapping.

## Discussion

### Observed reductions in blackfly-bite-per-capture estimates suggests mopHLCs are safer than sHLCs for onchocerciasis epidemiological monitoring

The primary objective of this study was to test the hypothesis that mopHLCs are safer than sHLCs for the capture of *O*. *volvulus* vectors. In order to do this we estimated the vector-capture-per-bite rates of the two vector capture techniques. Our experiments were conducted in a region just outside Amazonia onchocerciasis focus. In a previous study, performed as part of the WHO´s Onchocerciasis elimination programme, a total of 51,341 *O*. *volvulus* vectors were captured by sHLCs in the Amazonia onchocerciasis focus between 2006 and 2013 [7]. If mopHLCs had been used by these vector collectors, and they benefited from the same level of protection observed in our experiments, they could have avoided approximately 48,896 *O*. *volvulus* vector bites. Extrapolating globally, if the technique was used everywhere HLCs are presently being used for *O*. *volvulus* vector trapping, and provided similar protection to what we have observed, the technique could potentially help vector collectors avoid suffering millions of simuliid bites [1,2]. There is, thus, a strong argument for the utility of mopHLC to be tested in areas where HLCs are presently being used to monitor *O*. *volvulus* transmission and to launch studies to investigate if mopHLCs are equally effective for the capture of other *O*. *volvulus* vector species (most importantly *S*. *damnosum*) as they are for *S*. *oyapockense* capture. Whether the same potential health benefits are shared by highly experienced WHO vector collectors, who maybe better than our vector collectors at capturing vectors before they have the chance to bite [10], needs also to be investigated.

### Are mopHLCs a risk-free form of simuliid trapping?

While both sets of our bite-counting estimates clearly show that mopHLCs are safer than sHLCs, whether they completely eliminate (or can be adapted to completely eliminate) the risk of simuliid vector biting during simuliid trapping is not completely clear. In our study, the mopHLC bite-counter estimates made by our vector collectors (on the 28th of October 2017) were much lower than those made by our blind counters (2.125 $\bar{x}$ versus 7.9 $\bar{x}$). Although these results could be explained by the bite-counts performed by our vector collectors´ suffering from an observer bias, which our blind bite-counters did not suffer from, there are other alternative explanations too. Differences in the difficulty in distinguishing simuliid bite-marks from other types of skin blemishes on the days our two different bite counts were conducted, for example, could also explain this observation. The human immune response to blackfly bites can vary greatly across time and between individuals and this can affect the visual appearance of bite-marks as well as the ease with which they are identified [24–26]. As is illustrated in Figs 1–3, the simuliid bite marks counted in this study tended to be more pronounced immediately after our collections were completed, which may have made them easier to discriminate from other types of skin blemish in the first round of counting. Consistent with the notion that the first round of counts were more accurate and than the late counts, the standard deviation of almost all of the bite-count estimates made on the 28th of October can be seen to be much lower than those taken on the 30th (see Tables 1 and 2). It is, thus, likely that the lower mopHLC bite estimates taken on 28th of October more accurately reflect the true number of bites suffered by our vector collectors during the study than the counts made on 30th do.

As can been see in Tables 1 and 2, half of our vector collectors (JLC, CMA, JWPS) were scored by at least half of their 10 bite-counter assessors as having no bites at all on the legs they used in mopHLC trapping. And furthermore, two of these vector collectors (JLC, JWPS) were reported on the 28th of October 2017 (the date our standard deviation analysis suggest is more accurate) as having no bites at all on their legs by all four bite-counters that assessed them. It seems, thus, likely that one, if not several, of our vector collectors suffered no simuliid bites at all during their mopHLC trapping sessions. This observation is important because it suggests that at least some of the mopHLC trapping done in this study was completely simuliid-bite risk-free and thus that mopHLCs, if optimised, have the potential to become a risk-free strategy of simuliid vector trapping. At present, it is not clear to us if the vector collectors who were recorded (by between 8 and 9 of our bite-counters) as having simuliid bite marks on the legs, performed mopHLCs slightly differently from those that appeared to have suffered none. Although we attempted to train our vector collectors to apply an even amount of mineral oil across the surface of their legs, it may be, for example, that, in practise, they did not all prepare an equally thick and/or equally distributed layer of mineral oil across their legs. It could be, thus, that the low-levels of simuliid-biting suffered during mopHLC collections could be eliminated completely by adopting an optimised and standardised mineral oil skin-application protocol. It could, however, also be that the bite-marks on the legs of these vector collectors were acquired out-side of our designated collection periods. As noted in the methods section of this paper, although vector collectors were requested to use long-trousers whenever they were not collecting blackflies, wearing trousers in Equatorial São Gabriel da Cachoeira can be uncomfortable and, thus, this proved difficult to enforce.

### Does mopHLC trapping produce epidemiological data that is comparable with historic data?

Several human-bait-free trapping methods have been developed and used to monitor *O*. *volvulus* transmission in simuliids [8–13]. Whether these human-bait-free traps capture the exact same blackflies that are captured with HLCs and, thus, whether the *O*. *volvulus* transmission data generated from these traps can be treated as the same as data generated with HLCs has been questioned [9]. Given how important vector transmission data collected from HLCs has been for the design of past onchocerciasis disease control and elimination strategies, even small differences in the data generated from non-standard HLCs trapping could have non-trivial impacts on the effectiveness of future onchocerciasis control programmes [1–3]. Even though the methodology of our mopHLCs differs only slightly from sHLCs, we observed a significant difference in simuliid capture efficiency between it and sHLCs, which could translate into a difference in the epidemiological data collected by the two methods. Although, thus, methodological similarities between sHLCs and mopHLCs might intuitively lead one to expect the two techniques to obtain similar epidemiologically data this may not be the case and still needs to be shown; just as it has still needs to be shown if other simuliid host-seeking trapping methods like EWTs collect data that is epidemiological similar to that collected with sHLCs [8–10].

In addition to counting the number of simuliid vectors carrying L3 (infectious stage) *O*. *volvulus* larvae, epidemiological monitoring of onchocerciasis has traditionally also counted the number of parous biting female blackflies whenever possible. This counting of parous biting females, however, usually requires the collection of fresh specimens and thus it is not always practical to do. Although we did not specifically investigate the utility of mopHLCs for assessing whether females were parous or not, we believe that, because mopHLC trapping (like sHLC trapping) captures fresh blackfly specimens in a good physiological state, mopHLCs may be more reliable and/or convenient than EWTs for this purpose. We think that establishing whether this is the case should be viewed as a priority for anyone wishing to further develop mopHLC trapping as data from parous biting blackflies could be used to help characterise the differences between the epidemiological data generated from mopHLCs and sHLCs. And even if such differences prove to be significant, if they are well- characterised disease control planners should be able to factor them into their disease models to design reliable disease control strategies.

### Comparing sHLC and mopHLC trapping under different settings suggest that additional lures could assist mopHLC-only trapping

In our direct comparison experiments, mopHLC trapping captured simuliids significantly more efficiently than sHLC trapping; conversely, however, when mopHLC trapping was performed in isolation of sHLCs (and thus exposed skin trapping) mopHLC trapping was observed to capture simuliids significantly less efficiently than sHLCs. Our experiments have, thus, shown that while mopHLC performed in isolation of sHLCs can effectively trap *S*. *oyapockense* more safely than sHLCs, more mopHLC or longer mopHLCs will need to performed in order to acquire the same number of simuliids obtained with sHLCs. This loss of efficiency makes mopHLCs less practical for disease monitoring than sHLCs, it may, however, be possible to improve the efficiency of mopHLCs. While there is relatively little data on how anthropophilic simuliids are lured to human hosts, it is clear that skin odour volatiles play a crucial role in luring host-seeking anthropophilic mosquito species [27–29]. It could be, thus, that the loss of efficiency of mopHLC-only trapping, which we observed in our experiments, is explained by skin-applied mineral oil preventing or reducing the release of simuliid-attracting skin odour volatiles that are released in sHLCs. Consistent with this notion, recent human-bait-free simuliid trapping experiments performed in Africa, found that in addition to CO2 and various visual cues, sweaty socks and unwashed trousers, presumably soaked in skin odour volatiles, are a powerful attractant to lure for the African onchocerciasis vector *S*. *damnosum* [8,9]. It may, therefore, be possible to improve the efficiency of mopHLC by dressing vector collectors in clothes that prevent simuliid biting but allow the natural release of skin odour volatiles. It may also be possible to improve mopHLC using artificial lures like CO2 or lures (like sweaty socks) and/or combining these with specially clothed mopHLC vector collectors. Although such lures could potentially increase the yield of blackflies captured with mopHLC trapping, they could also potentially distort the epidemiological data collected from it. It is, therefore, important that if lures are used they are used with extreme care and the impact that they have on the epidemiological data they generate is carefully characterised.

### Other potential advantages of mopHLC blackfly trapping over the human-bait-free alternatives

While the greatest appeal of mopHLC trapping over the human-bait-free alternatives is that it is likely to generate epidemiological data that is similar to that generated by sHLCs, there are also potential logistical and financial factors that make it appealing too. For example, it can be difficult to transport and mount trapping apparatus used for non-human baited trapping to some field areas where blackfly trapping is needed, such as some heavily forested areas of the Amazonia onchocerciasis focus [15,17]. As the equipment used for mopHLC is extremely minimal is size, weight and cost, mopHLC trapping is logistically more practical than, for example, EWTs in such settings. Similarly, while it maybe possible to leave some non-human baited traps for very long periods (in order to the large numbers of vectors required for onchocerciasis monitoring), such long trapping periods could necessitate expensive and more frequent trips to hard-to-reach sites (i.e one trip for setting-up and one trip for dismounting of traps).

### Conclusions

Our results here have shown that mopHLCs can be used as an alternative to sHLCs and can significantly reduce (and may have to potential to eliminate) the number of bites suffered during human-baited *Simulium* trapping. Our results have also shown that while mopHLC trapping done in isolation of sHLCs are less efficient than sHLCs, there may be scope to improve their efficiency. Further research to investigate whether mopHLCs can eliminate health-risks to vector collectors is needed. Further research to determine if mopHLCs can provide health benefits to vector collectors capturing other *Simulium* vectors is also needed as are investigations into the health benefits of mopHLCs for more expert vector collectors who may be better than our vector collectors at capturing blackflies before they bite. We also believe, moreover, that further research in to the utility of the general approach of vector capture by surface (skin/hair/feather) application of viscous or sticky substances to living vertebrates for the capture of arthropod disease vectors also merits further research. And that the general approach described here could be extremely useful to other medical and veterinary arthropod borne disease research and control programmes.
